# Ca_3_[C_2_O_5_]_2_[CO_3_] is a pyrocarbonate which can be formed at *p*, *T*-conditions prevalent in the Earth’s transition zone

**DOI:** 10.1038/s42004-024-01293-1

**Published:** 2024-10-21

**Authors:** Dominik Spahr, Lkhamsuren Bayarjargal, Maxim Bykov, Lukas Brüning, Pascal L. Jurzick, Yu Wang, Victor Milman, Keith Refson, Mohamed Mezouar, Björn Winkler

**Affiliations:** 1 Goethe University Frankfurt, Institute of Geosciences, Altenhöferallee 1, 60438 Frankfurt, Germany; 2 Goethe University Frankfurt, Institute of Inorganic and Analytical Chemistry, Max-von-Laue-Straße 7, 60438 Frankfurt, Germany; 3 University of Cologne, Institute of Inorganic Chemistry, Greinstraße 6, 50939 Cologne, Germany; 4 Dassault Systèmes BIOVIA, 22 Cambridge Science Park, Cambridge, CB4 0FJ UK; 5 ISIS Facility, Science and Technology Facilities Council, Harwell Campus, Chilton, Didcot Oxon OX11 UK; 6 European Synchrotron Radiation Facility ESRF, 71 avenue des Martyrs, CS40220 38043 Grenoble Cedex 9, France

**Keywords:** Inorganic chemistry, X-ray diffraction, Solid-state chemistry, Chemical bonding, Geochemistry

## Abstract

Understanding the fate of subducted carbonates is a prerequisite for the elucidation of the Earth’s deep carbon cycle. Here we show that the concomitant presence of Ca[CO_3_] with CO_2_ in a subducting slab very likely results in the formation of an anhydrous mixed pyrocarbonate, ${{{{\rm{Ca}}}}}_{3}{\left[{{{{\rm{C}}}}}_{2}{{{{\rm{O}}}}}_{5}\right]}_{2}\left[{{{{\rm{CO}}}}}_{3}\right]$, at moderate pressure ( ≈ 20 GPa) and temperature ( ≈ 1500 K) conditions. We show that at these conditions ${{{{\rm{Ca}}}}}_{3}{\left[{{{{\rm{C}}}}}_{2}{{{{\rm{O}}}}}_{5}\right]}_{2}\left[{{{{\rm{CO}}}}}_{3}\right]$ can be obtained by reacting Ca[CO_3_] with CO_2_ in a laser-heated diamond anvil cell. The crystal structure was obtained from synchrotron-based single crystal X-ray diffraction data. Density Functional Perturbation Theory calculations in combination with experimental Raman spectroscopy results unambiguously confirmed the structural model. The crystal structure of ${{{{\rm{Ca}}}}}_{3}{\left[{{{{\rm{C}}}}}_{2}{{{{\rm{O}}}}}_{5}\right]}_{2}\left[{{{{\rm{CO}}}}}_{3}\right]$ is characterized by the presence of ${\left[{{{{\rm{CO}}}}}_{3}\right]}^{2-}$- and ${\left[{{{{\rm{C}}}}}_{2}{{{{\rm{O}}}}}_{5}\right]}^{2-}$-groups. The results presented here imply that the formation of ${{{{\rm{Ca}}}}}_{3}{\left[{{{{\rm{C}}}}}_{2}{{{{\rm{O}}}}}_{5}\right]}_{2}\left[{{{{\rm{CO}}}}}_{3}\right]$ needs to be taken into account when constructing models of the deep carbon cycle of the Earth.

## Abstract (web summary)

Carbonates are transported into the deep Earth by subduction of the oceanic lithosphere, but the stability fields of subducted carbonates as a function of pressure, temperature, and composition remain incompletely described. Here, the authors synthesize the anhydrous, mixed pyrocarbonate Ca3[C2O5]2[CO3] from Ca[CO3] and CO2 in a laser-heated diamond anvil cell at moderate pressure and elucidate its structural features.

## Introduction

Carbonates are the major host of carbon in the biosphere, the hydrosphere, in soils and in the Earth’s crust^1,2^. These carbonates are transported into the deep Earth by subduction of oceanic lithosphere, with an estimated total carbon transport of 40–60 Mt per year^3,4^. In order to understand the fate of subducted carbonates, their stability fields must be determined as a function of pressure, temperature, and composition. As Ca[CO_3_] and ${{{\rm{Ca}}}},{{{\rm{Mg}}}}{\left[{{{{\rm{CO}}}}}_{3}\right]}_{2}$ account for  >90% of the carbonates in the Earth’s crust^5^ their *p*, *T* phase diagrams haven been studied intensively^6,7^. Specifically, the *p*, *T*-phase diagram of Ca[CO_3_] has been studied numerous times, and at least 13 phases have been identified in the pressure range up to 50 GPa^6^, including some amorphous phases, and phases with dynamic disorder such as calcite-IV and calcite-V^8^.

Until recently, there was a consensus that for pressures prevalent in the Earth’s transition zone, i.e., down to a depth of 660 km and pressures up to 23 GPa (according to the preliminary reference Earth model^9^), the defining feature of the carbonates are ${\left[{{{{\rm{CO}}}}}_{3}\right]}^{2-}$ building blocks. Pressure-induced polymorphic transformations of calcite (Ca[CO_3_]), dolomite (${{{\rm{Ca}}}},{{{\rm{Mg}}}}{\left[{{{{\rm{CO}}}}}_{3}\right]}_{2}$), magnesite (Mg[CO_3_]) or siderite (Fe[CO_3_]), in which the coordination of the carbon atom changes from three-fold to four-fold were observed only at much higher pressures (*p* > 70 GPa) and temperatures, corresponding to lower mantle conditions^6,7,10,11^. Carbonates where the carbon is tetrahedrally coordinated will be denoted as *sp*^3^-carbonates in the following. However, more recently it was shown that the transformation from *sp*^2^-carbonates containing ${\left[{{{{\rm{CO}}}}}_{3}\right]}^{2-}$-groups to *sp*^3^-carbonates with [CO_4_]^4−^ building blocks can occur at significantly lower pressures in the presence of CO_2_, when instead of a polymorphic transition a reaction with CO_2_ may occur. This leads for example to the formation of ${{{{\rm{Ca}}}}}_{2}\left[{{{{\rm{C}}}}}_{4}{{{{\rm{O}}}}}_{10}\right]$-$I\bar{4}2d$ already at 34–45 GPa and 2000–3000 K^12^.

Alternatively, reactions of carbonates with CO_2_ at moderate pressures, such as are present in the Earth’s transition zone, may lead to the formation of pyrocarbonates, i.e. carbonates containing ${\left[{{{{\rm{C}}}}}_{2}{{{{\rm{O}}}}}_{5}\right]}^{2-}$ building blocks. Pyrocarbonates have been synthesized with alkali- and alkaline earth metal cations and with Pb^2+^- and Al^3+^-cations^13–17^. It is worthwhile to note here, that ${{{{\rm{Al}}}}}_{2}[{{{{\rm{C}}}}}_{2}{{{{\rm{O}}}}}_{5}]{[{{{{\rm{CO}}}}}_{3}]}_{2}$ is a pyrocarbonate containing ${\left[{{{{\rm{CO}}}}}_{3}\right]}^{2-}$ and ${\left[{{{{\rm{C}}}}}_{2}{{{{\rm{O}}}}}_{5}\right]}^{2-}$ building blocks^17^. This raises the question if pyrocarbonates with geologically relevant compositions, e.g., Ca[C_2_O_5_], exist, and, if so, if their stability field falls within a geologically relevant *p*, *T*-range. A further question would then be if they are more stable than aragonite^6^ or another high-pressure polymorph under geologically relevant conditions, such as high CO_2_-fugacities. There has been a prediction suggesting the stability of a calcium pyrocarbonate (with space group *Cc*), which was proposed to be the stable phase between 10 and 30 GPa, depending on temperature^18–20^, but no experimental evidence for the existence of this compound has been presented so far.

That inorganic pyrocarbonates have been obtained by reactions of a conventional carbonate with CO_2_^13–17^ is relevant for the Earth’s deep carbon cycle, where decarbonation and a corresponding release of CO_2_ into the surrounding mantle is expected in the vicinity of subducted slabs^21–23^. Inclusions of both Ca[CO_3_] and CO_2_ have been found in diamonds transported to the Earth’s surface from the mantle, supporting the relevance of this chemical system for the global carbon cycle^24–26^.

In the study presented here we investigated the system Ca[CO_3_]–CO_2_ in order to assess which calcium carbonates are likely to be stable at the Earth’s transition zone pressures in CO_2_-rich environments. From a general crystal chemical point of view, this study contributes to answering the question, whether pyrocarbonates can be synthesized for all cations for which conventional carbonates are known. The present study also serves as a benchmark for crystal structure prediction approaches^18–20^. Furthermore, obtaining a pyrocarbonate from the arguably most important conventional carbonate would further strengthen the hypothesis, that pyrocarbonates are ubiquitous in CO_2_-rich environments. It would also address the preliminary conclusion, based on the few pyrocarbonates which already have been obtained, that pyrocarbonates display a fascinating structural diversity, which in turn leads to the assumed chemical variability.

## Results and discussion

**Fig. 1:**
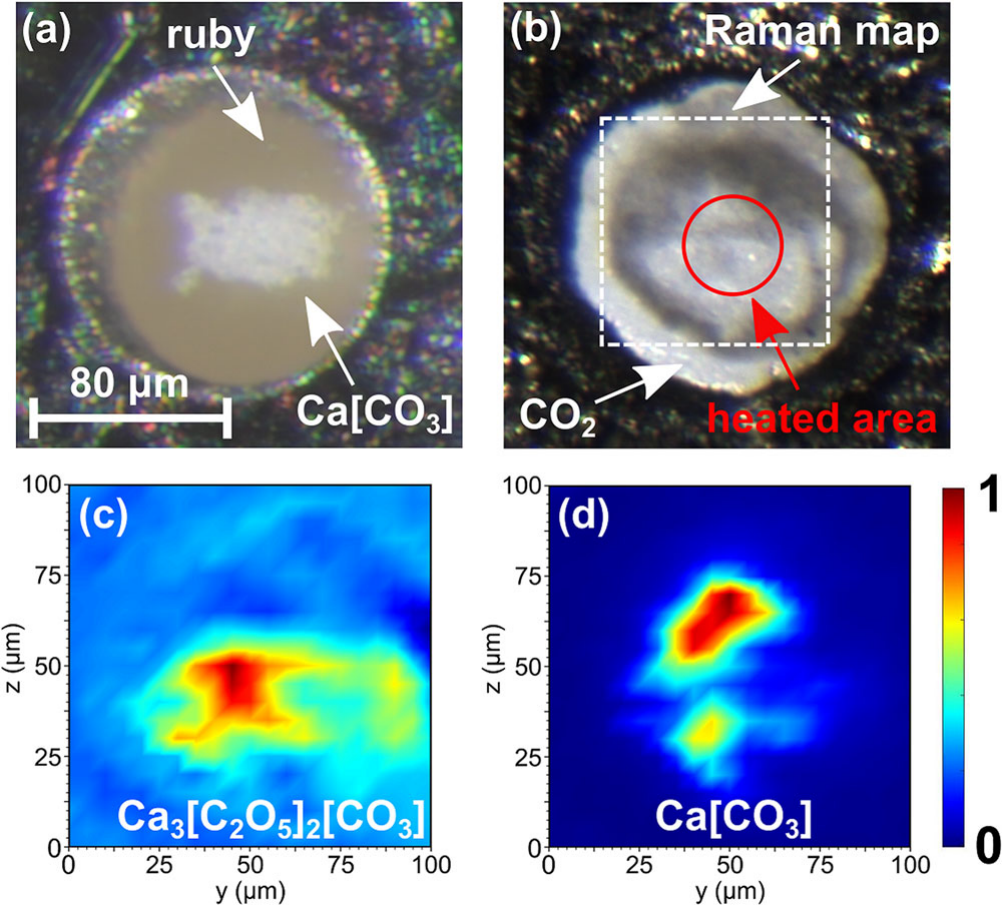
Raman maps of ${{{{\rm{Ca}}}}}_{3}{\left[{{{{\rm{C}}}}}_{2}{{{{\rm{O}}}}}_{5}\right]}_{2}\left[{{{{\rm{CO}}}}}_{3}\right]$ and Ca[CO_3_] after their synthesis. **a** Compacted Ca[CO_3_] (calcite) powder and ruby chip in the gasket hole of the DAC before the cryogenic loading. **b** Ca[CO_3_] + CO_2_ mixture after the cryogenic loading and laser heating to  ≈1500(200) K at 20(2) GPa. The position of the Raman map is indicated by a dashed square. **c** Raman map of ${{{{\rm{Ca}}}}}_{3}{\left[{{{{\rm{C}}}}}_{2}{{{{\rm{O}}}}}_{5}\right]}_{2}\left[{{{{\rm{CO}}}}}_{3}\right]$ (≈1050 cm^−1^). **d** Raman map of unreacted Ca[CO_3_] (≈1150 cm^−1^).

**Fig. 2:**
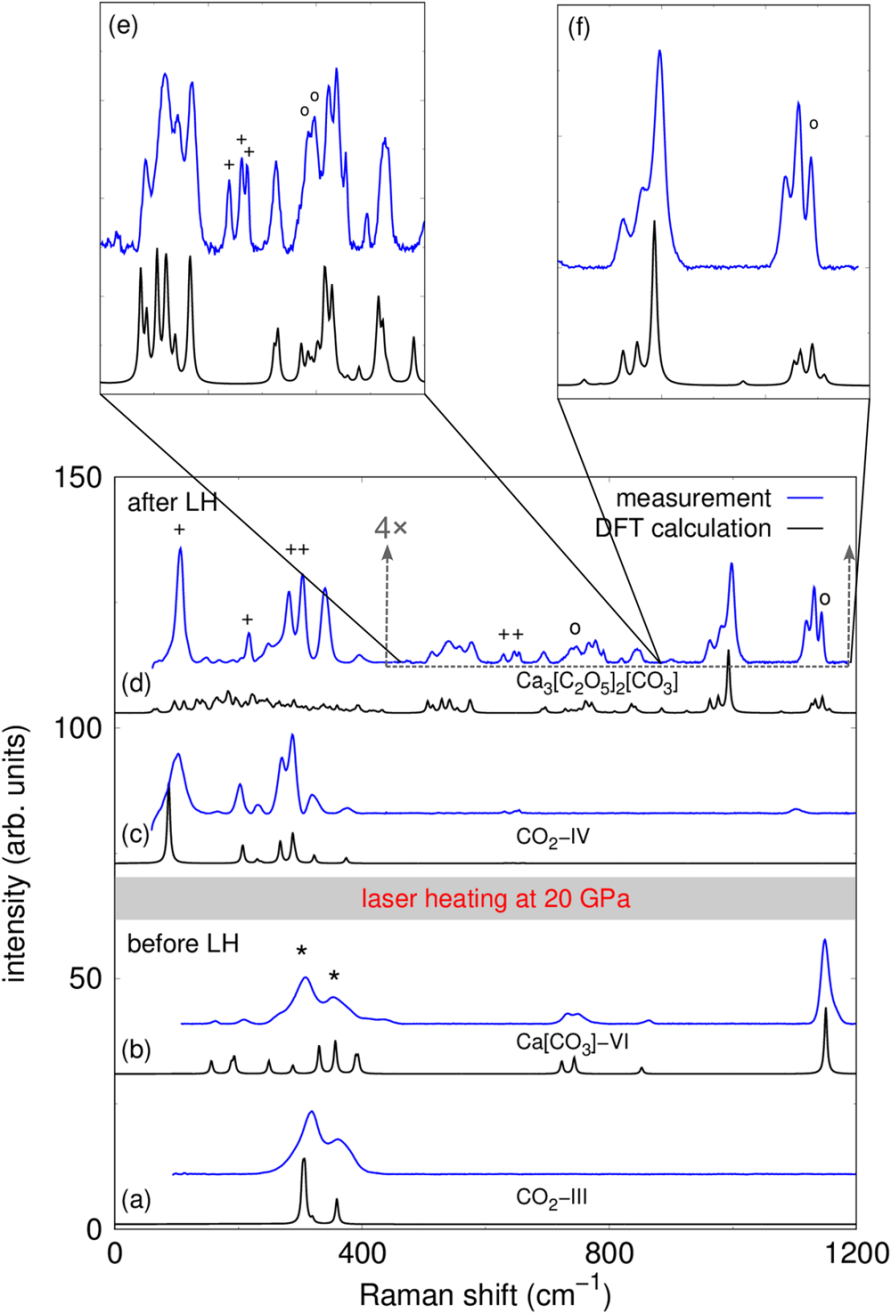
Experimental and calculated Raman spectra before and after the synthesis of ${{{{\rm{Ca}}}}}_{3}{\left[{{{{\rm{C}}}}}_{2}{{{{\rm{O}}}}}_{5}\right]}_{2}\left[{{{{\rm{CO}}}}}_{3}\right]$. **a** Raman spectra for CO_2_-III at 20(2) GPa. **b** Raman spectra for Ca[CO_3_]-VI at 20(2) GPa. **c** Raman spectra of CO_2_-IV after laser heating. **d** Raman spectra of ${{{{\rm{Ca}}}}}_{3}{\left[{{{{\rm{C}}}}}_{2}{{{{\rm{O}}}}}_{5}\right]}_{2}\left[{{{{\rm{CO}}}}}_{3}\right]$ after the synthesis at elevated pressures and temperatures. The intensity of the experimental Raman data was upscaled by factor four in the region between 450 and 1200 cm^−1^, to account for the strong Raman modes of CO_2_-IV at low wavenumbers. Close-up of the region between **e** 450–900 cm^−1^ and **f** 900–1200 cm^−1^. Experimental Raman spectra are shown in blue, DFT-based calculations are shown in black. The Raman shifts of the theoretical spectra were scaled by 4% to account for the well-established DFT-GGA-PBE underbinding. Peaks of CO_2_-III are marked by an asterisk (*), of CO_2_-IV by a cross ( + ) and of Ca[CO_3_] by a circle (∘).

The high-pressure experiments were carried out in laser-heated diamond anvil cells (LH-DACs). The DACs were loaded with Ca[CO_3_] (calcite) and a ruby chip for pressure determination^27^ (Fig. 1a). Afterwards, CO_2_-I (dry ice) was added by cryogenic loading. Finally, the Ca[CO_3_]–CO_2_ mixture was compressed to the target pressure without intermediate heating. Cold compressing CO_2_-I ($Pa\bar{3}$) causes a pressure-induced phase transition to CO_2_-III (*Cmca*) in a broad (≈5 GPa) pressure range around  ≈12 GPa^28,29^. At 20(2) GPa the experimental Raman spectrum is in very good agreement with the Raman spectrum of CO_2_-III obtained from the DFT-based calculations (Fig. 2a). Above 15 GPa the metastable polymorph Ca[CO_3_]-VI is present at low temperatures^6^. Our experimental Raman spectrum at 20(2) GPa, prior to the laser heating, is accurately reproduced by the DFT-calculated Raman spectrum for Ca[CO_3_]-VI with $P\bar{1}$ space group symmetry (Fig. 2b)^30^.

At pressures of 15, 20, and 25 GPa Ca[CO_3_] was laser-heated in the CO_2_ atmosphere from both sides (Fig. 1b). Raman spectroscopy at ambient temperature was employed to monitor the progress of the reaction induced by the laser-heating. At these pressures the direct and indirect heating of CO_2_ causes the appearance of the high-temperature polymorphs CO_2_-II and CO_2_-IV (Fig. 2c and “Methods” section)^31,32^. In addition, Raman spectroscopy, carried out after cold compression and laser heating indicated that between 15 and 25 GPa a reaction between Ca[CO_3_] and CO_2_ had occurred. New Raman modes appear in the region between 700 cm^−1^ and 1050 cm^−1^ at ambient temperatures (Fig. 2d), which are characteristic for vibrations of ${\left[{{{{\rm{C}}}}}_{2}{{{{\rm{O}}}}}_{5}\right]}^{2-}$-groups^13–17^. Spatially resolved Raman spectroscopy was employed to locate the product phase in the DAC (Fig. 1c) and to identify the region were unreacted Ca[CO_3_] remained (Fig. 1d). This was the basis to efficiently locate crystals of the new carbonate in subsequent synchrotron-based diffraction experiments.

**Fig. 3:**
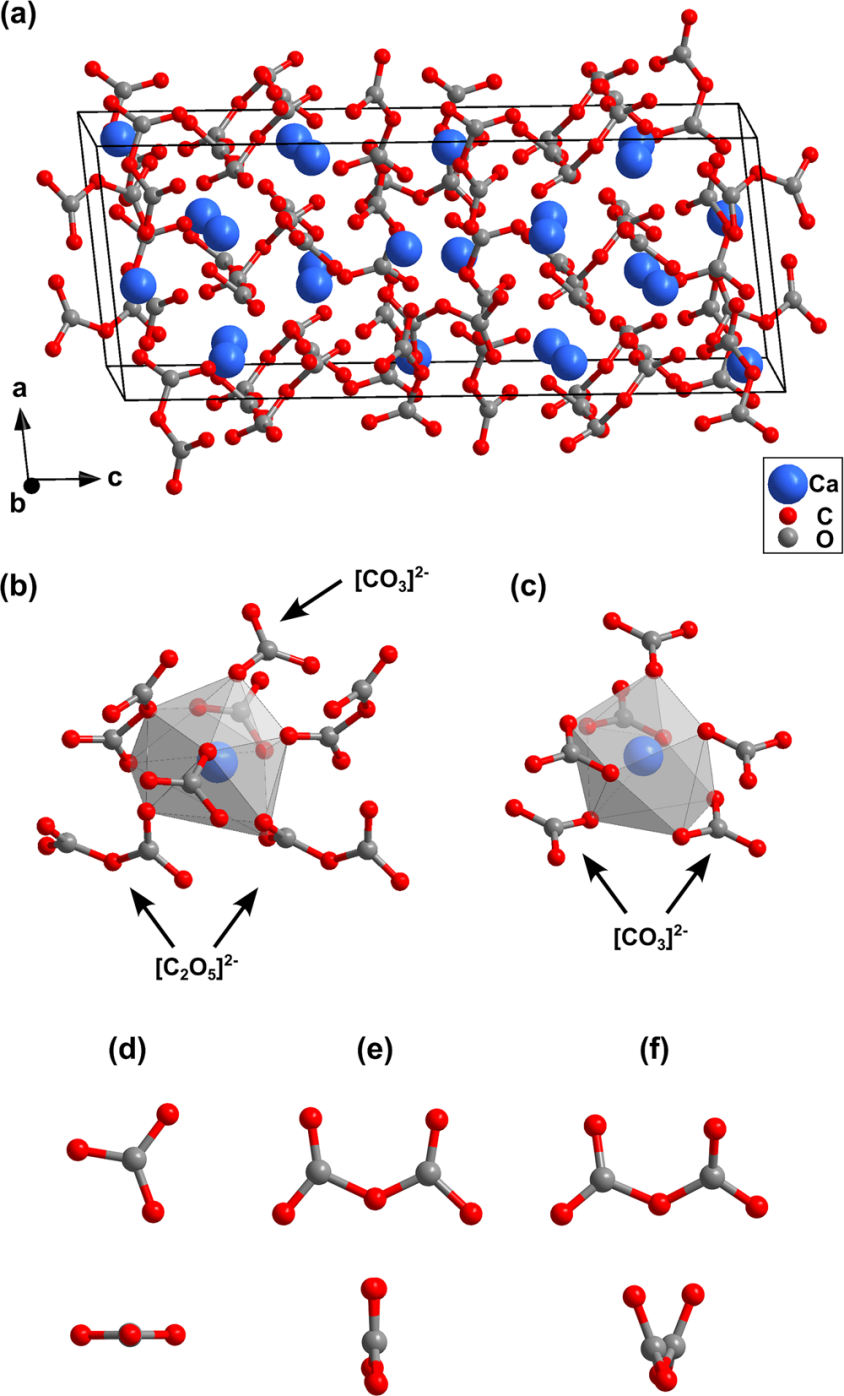
Crystal structure of ${{{{\rm{Ca}}}}}_{3}{\left[{{{{\rm{C}}}}}_{2}{{{{\rm{O}}}}}_{5}\right]}_{2}\left[{{{{\rm{CO}}}}}_{3}\right]$ and geometries of its constituent units. **a** Monoclinic structure (*P*2_1_/*n*, *Z* = 8) of calcium pyrocarbonate (${{{{\rm{Ca}}}}}_{3}{\left[{{{{\rm{C}}}}}_{2}{{{{\rm{O}}}}}_{5}\right]}_{2}\left[{{{{\rm{CO}}}}}_{3}\right]$) obtained from synchrotron X-ray single crystal structure solution at 20(2) GPa viewed along the *b*-axis. **b** Coordination polyhedron (gray) of a selected Ca^2+^-cation (blue sphere) in the crystal structure of ${{{{\rm{Ca}}}}}_{3}{\left[{{{{\rm{C}}}}}_{2}{{{{\rm{O}}}}}_{5}\right]}_{2}\left[{{{{\rm{CO}}}}}_{3}\right]$. **c** Coordination polyhedron of a selected Ca^2+^-cation in the aragonite crystal structure, which is the stable Ca[CO_3_] polymorph in the absence of further phases at transition zone conditions^6,35^. Geometry of selected **d** ${\left[{{{{\rm{CO}}}}}_{3}\right]}^{2-}$- and **e**, **f** ${\left[{{{{\rm{C}}}}}_{2}{{{{\rm{O}}}}}_{5}\right]}^{2-}$-groups in ${{{{\rm{Ca}}}}}_{3}{\left[{{{{\rm{C}}}}}_{2}{{{{\rm{O}}}}}_{5}\right]}_{2}\left[{{{{\rm{CO}}}}}_{3}\right]$.

The results from our synchrotron-based single crystal X-ray diffraction measurements show that the unknown phase is a novel calcium carbonate, which was formed at pressures prevalent in the Earth’s transition zone. The crystal structure was solved from the temperature-quenched reaction product at 20(2) GPa. The novel phase is a mixed pyrocarbonate with ${{{{\rm{Ca}}}}}_{3}{\left[{{{{\rm{C}}}}}_{2}{{{{\rm{O}}}}}_{5}\right]}_{2}\left[{{{{\rm{CO}}}}}_{3}\right]$ composition hosting ${\left[{{{{\rm{CO}}}}}_{3}\right]}^{2-}$- and ${\left[{{{{\rm{C}}}}}_{2}{{{{\rm{O}}}}}_{5}\right]}^{2-}$-groups. Its crystal structure is complex (Fig. 3a). ${{{{\rm{Ca}}}}}_{3}{\left[{{{{\rm{C}}}}}_{2}{{{{\rm{O}}}}}_{5}\right]}_{2}\left[{{{{\rm{CO}}}}}_{3}\right]$ crystallizes at 20(2) GPa in the monoclinic space group *P*2_1_/*n* with *Z* = 8 and the structure was solved with a *R*-value of 7.8% and a reflection to parameter ratio of 10:1 (Supplementary Discussion and Supplementary Table 1). The unit cell is elongated in *c*-direction (*a* = 8.020(4) Å, *b* = 8.8450(7) Å, *c* = 20.616(12) Å and *β* = 96.18(6)°) and contains 168 atoms. This is a new structure type. The new structure is characterized by the presence of both, isolated ${\left[{{{{\rm{CO}}}}}_{3}\right]}^{2-}$ and ${\left[{{{{\rm{C}}}}}_{2}{{{{\rm{O}}}}}_{5}\right]}^{2-}$ building blocks, without any residues attached to the oxygen atoms (Fig. 3b). The ${\left[{{{{\rm{CO}}}}}_{3}\right]}^{2-}$-groups are nearly trigonal-planar (Fig. 3d). In contrast, the ${\left[{{{{\rm{C}}}}}_{2}{{{{\rm{O}}}}}_{5}\right]}^{2-}$-groups occur in different geometries, with different rotation angles (5°, 32°, 41°, and 46°) around the bridging oxygen atom between the two connected ${\left[{{{{\rm{CO}}}}}_{3}\right]}^{2-}$-groups (Fig. 3e, f). These geometries are in good agreement with the observed flexibility of the ${\left[{{{{\rm{C}}}}}_{2}{{{{\rm{O}}}}}_{5}\right]}^{2-}$-group in other pyrocarbonates^13–17^. The appearance of ${\left[{{{{\rm{CO}}}}}_{3}\right]}^{2-}$- and ${\left[{{{{\rm{C}}}}}_{2}{{{{\rm{O}}}}}_{5}\right]}^{2-}$-groups together was also observed in the crystal structure of ${{{{\rm{Al}}}}}_{2}[{{{{\rm{C}}}}}_{2}{{{{\rm{O}}}}}_{5}]{[{{{{\rm{CO}}}}}_{3}]}_{2}$, but here only planar ${\left[{{{{\rm{C}}}}}_{2}{{{{\rm{O}}}}}_{5}\right]}^{2-}$-groups are present^17^. Due to the polymerization of the ${\left[{{{{\rm{CO}}}}}_{3}\right]}^{2-}$-groups in ${{{{\rm{Ca}}}}}_{3}{\left[{{{{\rm{C}}}}}_{2}{{{{\rm{O}}}}}_{5}\right]}_{2}\left[{{{{\rm{CO}}}}}_{3}\right]$ this new phase hosts a significantly higher carbon content (>20%) per unit cell volume than known Ca[CO_3_] phases such as aragonite or calcite VII at the same pressure^6,33,34^.

After the structure determination of ${{{{\rm{Ca}}}}}_{3}{\left[{{{{\rm{C}}}}}_{2}{{{{\rm{O}}}}}_{5}\right]}_{2}\left[{{{{\rm{CO}}}}}_{3}\right]$, we employed the experimental structural model as a trial structure for DFT-based model calculations. As expected, a full geometry optimization resulted in a structural model which nicely reproduces our experimental data (Supplementary Table 1). Due to the low monoclinic symmetry (*P*2_1_/*n*), the large unit cell (*V* = 1454(1) Å^3^, 168 atoms), and a unit cell shape where the *a*- and *b*-axes are only about half as long as the *c*-axis, the comparison of a computed Raman spectrum to an experimentally determined one required significant computational resources. Nevertheless, the theoretical Raman spectrum unambiguously confirmed that the structure solution was correct (Fig. 2d). The enlargements of the Raman spectra in the region between 450–900 cm^−1^ (Fig. 2d) and 900–1200 cm^−1^ (Fig. 2f) show that the DFPT spectra faithfully reproduce the intensities of the Raman bands of the new carbonate.

**Fig. 4:**
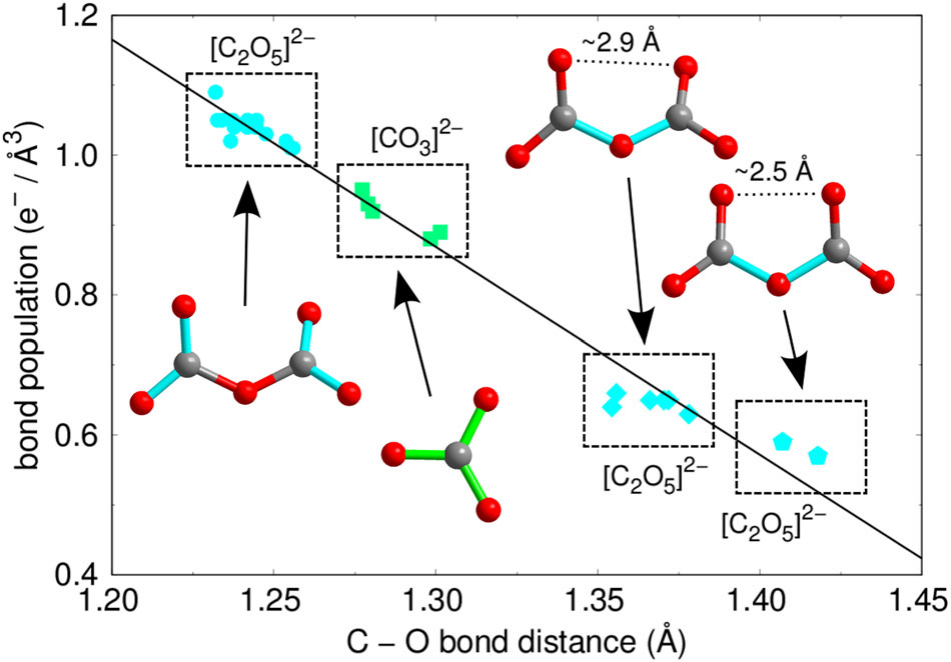
Bond population of the C–O bonds in ${{{{\rm{Ca}}}}}_{3}{\left[{{{{\rm{C}}}}}_{2}{{{{\rm{O}}}}}_{5}\right]}_{2}\left[{{{{\rm{CO}}}}}_{3}\right]$. Mulliken bond population analysis of C–O bonds in the ${\left[{{{{\rm{CO}}}}}_{3}\right]}^{2-}$ (green) and ${\left[{{{{\rm{C}}}}}_{2}{{{{\rm{O}}}}}_{5}\right]}^{2-}$ (blue) building blocks of ${{{{\rm{Ca}}}}}_{3}{\left[{{{{\rm{C}}}}}_{2}{{{{\rm{O}}}}}_{5}\right]}_{2}\left[{{{{\rm{CO}}}}}_{3}\right]$ from DFT calculations. The solid line is a linear fit to the theoretical data.

From their Mulliken population, the strengths of the C–O bonds in the ${\left[{{{{\rm{CO}}}}}_{3}\right]}^{2-}$- and ${\left[{{{{\rm{C}}}}}_{2}{{{{\rm{O}}}}}_{5}\right]}^{2-}$-groups can be estimated. Bonds between a carbon atom and a terminal oxygen in a ${\left[{{{{\rm{C}}}}}_{2}{{{{\rm{O}}}}}_{5}\right]}^{2-}$-group are strongest, those between the carbon and the oxygen atoms in a ${\left[{{{{\rm{CO}}}}}_{3}\right]}^{2-}$-groups are intermediate, while bonds between carbon atoms and the central oxygen in a ${\left[{{{{\rm{C}}}}}_{2}{{{{\rm{O}}}}}_{5}\right]}^{2-}$-group have the lowest Mulliken populations (Fig. 4). In addition, the Mulliken bond population analysis shows that the Mulliken bond population of C–O bonds in ${{{{\rm{Ca}}}}}_{3}{\left[{{{{\rm{C}}}}}_{2}{{{{\rm{O}}}}}_{5}\right]}_{2}\left[{{{{\rm{CO}}}}}_{3}\right]$ linearly depends on the C–O bond distance. The third building block in the ${{{{\rm{Ca}}}}}_{3}{\left[{{{{\rm{C}}}}}_{2}{{{{\rm{O}}}}}_{5}\right]}_{2}\left[{{{{\rm{CO}}}}}_{3}\right]$ crystal structure, in addition to the [CO_3_]- and [C_2_O_5_]-groups, are Ca–O-polyhedra. The coordination of the calcium atoms ranges from 9 to 11 oxygen atoms (Supplementary Fig. 1). The coordination polyhedra are irregular, with Ca–O bonds between 2.2 Å and 2.9 Å. The [CaO_9_]-polyhedra (Fig. 3b) are similar to those in aragonite (Fig. 3c), the stable Ca[CO_3_] polymorph in the absence of further phases at transition zone conditions^6,35^.

After we established that the DFT structural model is an accurate representation of the new phase, we computed the pressure dependence of the unit cell (Supplementary Fig. 2). The calculations show that for this phase, a van der Waals correction to the standard DFT-GGA-PBE approach is required, as otherwise the pressure dependence of the unit cell volumes at low pressures cannot be described with a reasonable EoS. At pressures *p* > 10 GPa the van der Waals corrected unit cell volumina are only slightly smaller than those obtained without the correction, and hence the latter have been employed for the DFPT calculations. The pressure-induced change of the unit cell volume is as expected at pressures *p* ≥ 5 GPa, and we fitted a 3rd-order Birch–Murnaghan equation of states (EoS) to unit cell volume derived from the calculations for these pressures to obtain the theoretical bulk modulus (*K*_0_)^36,37^. We obtained a bulk modulus of *K*_0_ = 60(1) GPa with a pressure derivative of *K*_p_ = 5.2(1) between 5 and 35 GPa. The bulk modulus is slightly higher than those computed for other pyrocarbonates^13–16^.

The computed pressure dependence of the unit cell volume for pressures from ambient up to 5 GPa cannot be reasonably described by an extrapolation of the high pressure behavior, and seems to imply a structural instability. However, understanding this behavior by computing the lattice dynamics or elastic stiffness coefficients, would require computational resources significantly beyond those that were available and as this behavior is of little relevance to the questions addressed in the present study, this aspect has not been investigated further. A structural instability of ${{{{\rm{Ca}}}}}_{3}{\left[{{{{\rm{C}}}}}_{2}{{{{\rm{O}}}}}_{5}\right]}_{2}\left[{{{{\rm{CO}}}}}_{3}\right]$ at pressures  <5 GPa is supported by our experimental Raman data. The characteristic Raman modes of ${{{{\rm{Ca}}}}}_{3}{\left[{{{{\rm{C}}}}}_{2}{{{{\rm{O}}}}}_{5}\right]}_{2}\left[{{{{\rm{CO}}}}}_{3}\right]$, in the region between 450 and 1200 cm^−1^, can only be observed down to 5(1) GPa during decompression of the DAC (Supplementary Fig. 3). At ambient conditions only the Raman modes of calcite (Ca[CO_3_]) were measured in the gasket hole after pressure release (Supplementary Fig. 3).

There have been many crystal structure prediction studies aimed at predicting the structure of Ca-carbonates at high pressures and high temperatures^18,38,39^. To the best of our knowledge, no crystal structure prediction study indicated that pyrocarbonates may be potential high-pressure carbonate phases before the first experimental study in 2022 yielded an inorganic anhydrous pyrocarbonate^14^. Specifically, the crystal structure prediction of high-pressure phases in the system CaO–CO_2_ in the pressure range from 0 to 100 GPa by Sagatova et al.^38^ did not point towards the existence of a calcium pyrocarbonate. In a later study it was predicted that a monoclinic (space group *Cc*) pyrocarbonate with composition Ca[C_2_O_5_] would be stable between 10 and 30 GPa, depending on temperature^18^, but this compound has no resemblance to the compound found here. The assumption that the reaction of Ca[CO_3_] with CO_2_ leads to Ca[C_2_O_5_]-*Cc* was the basis of a computational study linking the elastic behavior of carbonates to seismic anomalies^19^, and this assumption seems to be problematic based on our experimental findings.

The determination of the relative stability of ${{{{\rm{Ca}}}}}_{3}{\left[{{{{\rm{C}}}}}_{2}{{{{\rm{O}}}}}_{5}\right]}_{2}\left[{{{{\rm{CO}}}}}_{3}\right]$ with respect to aragonite, to the *sp*^3^-carbonate ${{{{\rm{Ca}}}}}_{2}\left[{{{{\rm{C}}}}}_{4}{{{{\rm{O}}}}}_{10}\right]$-$I\bar{4}2d$^12^, to Ca[CO_3_]-VII^34^ and to the hypothetical pyrocarbonate Ca[C_2_O_5_]-*Cc*^18^ in the presence of CO_2_ is challenging, as it involves a reaction with molten CO_2_, and hence, from a computational point of view, computed reaction enthalpies are very likely only of limited accuracy. Due to the complexity of the structure, computations even in the quasi-harmonic approximation would require enormous computational resources. In the athermal limit, and using either CO_2_-III or CO_2_-IV, the compound obtained here would be more stable than a mechanical mixture of aragonite and CO_2_. It would, however, be not more stable than a mechanical mixture of the *Cc*-phase predicted by Sagatova et al.^18^, CaO and CO_2_. However, the latter result is not supported by our experimental findings, and hence we conclude that the underlying assumption, namely that the athermal limit is a sufficiently accurate approximation, is incorrect.

## Conclusion

In summary, the present study has answered the question, whether a calcium pyrocarbonate can be synthesized and which structure this phase would have. The combination of a structure solution by single crystal X-ray diffraction and the reproduction of the the experimental Raman spectrum by DFPT calculations has led to an unambiguous structural model for the mixed carbonate-pyrocarbonate ${{{{\rm{Ca}}}}}_{3}{\left[{{{{\rm{C}}}}}_{2}{{{{\rm{O}}}}}_{5}\right]}_{2}\left[{{{{\rm{CO}}}}}_{3}\right]$, which bears no resemblance to a structural model predicted for a pyrocarbonate earlier^18^. We show that structure determination for a low-symmetry compound with more than 150 atoms in a unit cell is best achieved using experimental techniques, using theoretical analysis only as a supplementary tool. The formation conditions of the new phase make it likely that it is present in the Earth’s transition zone in subducting slabs, as these, during devolatilisation, would provide both the carbonate and the CO_2_ required.

## Methods

### Sample material

We used commercial calcite (Ca[CO_3_]) powder (99.95% purity, Merck KGaA, Darmstadt, Germany) for the high-pressure experiments without further purification. Before the DAC loading the Ca[CO_3_] powder was dried in an oven at 573(1) K for 12 h. Afterwards, the powder was compacted between a diamond and a glass plate to obtain a 10–20 μm thin powder compact. We used the CO_2_ gas for the gas-jet (Nippon gases, purity ≥99.995%) and the argon purge gas (Nippon gases, purity ≥99.999%) as purchased.

### High-pressure experiments

The high-pressure experiments were carried out using Boehler-Almax type DACs with 350 μm culet size^40^. Diamonds with 70° opening angle on both sides were used for the experiments. Re-gaskets were pre-indented to a thickness of  ≈50 μm and placed between the top and bottom diamonds. Gasket-holes with 110 μm diameter were drilled by a custom-built laser setup. In a first step the powder compact with dimensions of  ≈70 × 30 μm^2^ and a thickness of 10–20 μm was placed on the culet of the bottom diamond. Afterwards, we added a ruby chip for pressure determination in the gasket hole on the bottom diamond. The pressure was determined by measuring the shift of the ruby fluorescence and we assume an error of 6% due to non-hydrostatic conditions^27^. We assume that the pressure conditions in the DAC before laser-heating are very likely non-hydrostatic as CO_2_-III may sustain pressure gradients up to 0.2 GPa μm^−1^ at high pressures without heating^41^.

The CO_2_ (dry-ice) was directly condensed into the gasket hole using a custom-built cryogenic loading system^16^ derived from an earlier concept^42^. The DAC was slightly opened and placed on a liquid nitrogen cooled Cu-holder and it was cooled down to  ≈100 K. We used a small nozzle to align the CO_2_ gas jet with 5 l min^−1^ directly on the gap between upper diamond and the gasket. Ar (10 l min^−1^) was used as a purge-gas to avoid the precipitation of H_2_O ice. The precipitation of the CO_2_ in the gasket hole was monitored by an optical microscope and a camera. After a sufficient amount of CO_2_ was gathered in the gasket hole, the DAC was tightly closed and compressed to the target pressure without intermediate heating.

### Raman spectroscopy and laser heating

High-pressure Raman spectroscopy and the double-sided laser-heating in DACs were performed using a custom-built set-up^6^. Raman spectroscopy was performed with an Oxxius LCX-532S Nd:YAG laser (*λ* = 532.14 nm) in combination with a Princeton Instruments ACTON SpectraPro (SP-2356) spectrograph equipped with a Pixis 256E CCD camera. Applying a laser power of 250 mW on the sample, the spot size of the Raman laser was  ≈6 μm. Raman maps were measured on a grid with a step-size of 5 μm in *x*- and *y*-direction. Afterwards, the background was corrected using the software package Fityk^43^.

Double-sided laser-heating was performed using a Coherent Diamond K-250 pulsed CO_2_ laser (*λ* = 10,600 nm). The laser power was adjusted to achieve a coupling of the laser to the sample using a laser power of 1–3 W. Focusing on the sample results in a heating area of  ≈60 × 60 μm^2^ and the highest temperature achieved during the laser heating was ${T}_{\max }\approx 1500(200)$ K. The temperatures were determined by the two-color pyrometer method, employing Planck and Wien fits^44^. The heating time was  ≈ 60 min. It is well established that laser-heating in DACs always suffers from large temperature gradients and the actual temperature is strongly dependent on the coupling of the laser with the sample, especially at lower temperatures. We estimate an uncertainty of at least  ±10% of the nominal temperature in the laser-heated region depending on the focus of the laser beam, based on typical 2D temperature-gradient determination experiments performed in DACs^45^.

**Fig. 5:**
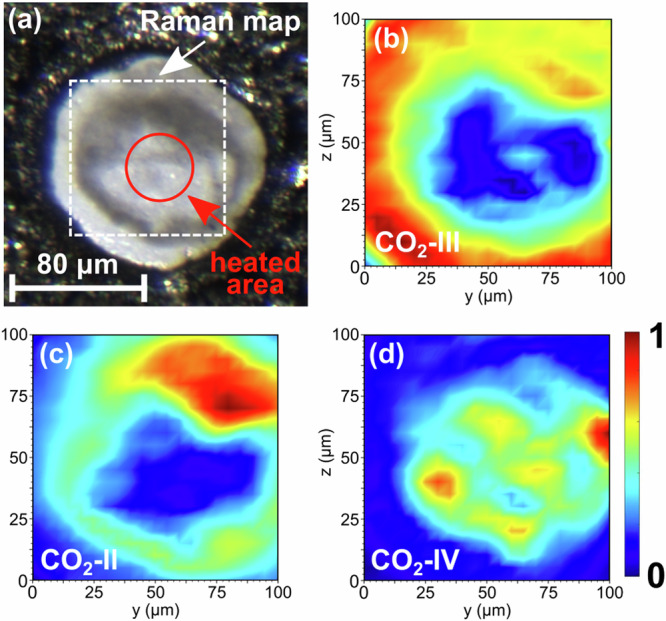
Raman maps showing the distribution of CO_2_-phases after laser heating. **a** The Ca[CO_3_] + CO_2_ mixture after laser heating at 20(2) GPa up to temperatures of  ≈1500(200) K. The position of the Raman map is indicated by a dashed square. Raman maps of: **b** CO_2_-III, **c** CO_2_-II, and **d** CO_2_-IV.

At low pressures (<40 GPa) several different phases are present in the CO_2_ phase diagram, depending on the temperature^46^. In our experiments the temperature gradient in the gasket hole during the laser heating of the CO_2_ at  ≈20 GPa causes the appearance of the high-temperature polymorphs phase II^31^ (Fig. 5c) and IV^32^ (Fig. 5d). In addition, the low temperature phase III^28^ is still present in the unheated regions (Fig. 5b).

### Single crystal synchrotron X-ray diffraction

Single crystal synchrotron X-ray diffraction had been carried out at the ESRF in Grenoble, France, at the high pressure beam line ID27^47^. The beam size on the sample was  ≈2 × 2 μm^2^ (FWHM), focused by Kirkpatrick Baez mirrors. The diffraction data were collected using an Eiger2 X 9M CdTe detector, a wavelength of 0.3738 Å (33.2 keV) and a detector to sample distance of 183 mm. We rotated the DAC by  ±33° around the vertical axis perpendicular to the beam while collecting frames in 0.5° steps with 2 s acquisition time per frame.

The detector to sample distance was calibrated from the powder diffraction of a CeO_2_ standard and using the software DIOPTAS^48^. The diffractometer/detector geometry for the analysis of the single crystal diffraction data was calibrated using diffraction data collected from a single crystal of enstatite in a DAC at ambient pressure. After the data collection, the reflections were indexed and integrated employing CrysAlis^PRO^ (version 43.67a)^49^. We used the Domain Auto Finder program (DAFi) to find possible single crystal domains for the subsequent data reduction^50^. The structure solution and refinement were performed using the software package OLEX2 employing SHELXT for the crystal structure determination and and SHELXL for the refinement^51–53^.

**Fig. 6:**
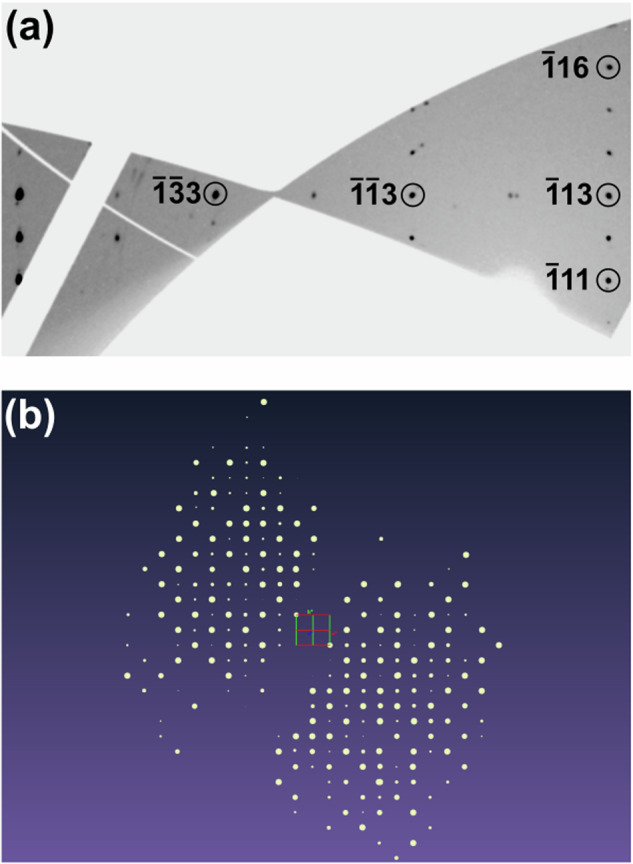
Unwarped image of the raw-experimental data and schematic depiction of the reflections in reciprocal space. **a** *Unwarped* image of the raw-experimental data after data reduction. The ($\bar{1}kl$) area is shown. Other reflections are due to other phases/domains present in the sample chamber. **b** Schematic depiction of the reflections in reciprocal space using the Ewald-Explorer in CrysAlis after data reduction and later used for the refinement. Projection of the reciprocal space is shown along c^*^.

Figure 6a shows a part of an *unwarped* image of the raw-experimental data after processing of the ($\bar{1}kl$) area in CrysAlis. Besides the reflection of the new phase, reflections and powder rings of different CO_2_ phases, Ca[CO_3_] and diamond can be found in the diffraction data. Nevertheless, the data quality is very good for high-pressure measurement performed in a DAC on a multi-grain reaction product. A projection of the reciprocal space shows the distribution of reflections and the effect of the shading of diffracted beams due to the DAC after data reduction (Fig. 6 b). The coverage of the reciprocal space is sufficient for a subsequent single crystal structure solution.

### Density functional theory-based calculations

First-principles calculations were carried out within the framework of density functional theory (DFT), employing the Perdew–Burke–Ernzerhof (PBE) exchange-correlation functional and the plane wave/pseudopotential approach implemented in the CASTEP simulation package^54–56^. “On the fly” norm-conserving or ultrasoft pseudopotentials generated using the descriptors in the CASTEP data base were employed in conjunction with plane waves up to a kinetic energy cutoff of 1020 eV or 630 eV, for norm-conserving and ultrasoft pseudopotentials, respectively. The accuracy of the pseudopotentials is well established^57^. A Monkhorst–Pack grid was used for Brillouin zone integrations^58^. We used a distance between grid points of  <0.023 Å^−1^. Convergence criteria for geometry optimization included an energy change of  <5 × 10^−6^ eV atom^−1^ between steps, a maximal force of  <0.008 eV Å^−1^ and a maximal component of the stress tensor  < 0.02 GPa. Phonon frequencies were obtained from density functional perturbation theory (DFPT) calculations^59,60^. Raman intensities were computed using DFPT with the “2*n* + 1” theorem approach^61^. A correction scheme for van der Waals (v.d.W.) interactions was applied for investigating the compression behavior by DFT-calculations. We employed the correction scheme developed by Tkatchenko and Scheffler^62^.

## Supplementary information

Supplementary Material

## Data Availability

The X-ray crystallographic coordinates for the structure reported in this study has been deposited at the Cambridge Crystallographic Data Centre (CCDC), under deposition numbers 2343767 (single crystal) 2343768 (DFT-calculation). These data can be obtained free of charge from The Cambridge Crystallographic Data Centre via www.ccdc.cam.ac.uk/data_request/cif. The supplementary material contains additional information to the results of the single crystal structure solution, Raman spectroscopy and DFT-based calculations.
