# Supplementary material for: Ca3[C2O5]2[CO3] is a pyrocarbonate which can be formed at p, T-conditions prevalent in the Earth’s transition zone
Source: Commun Chem. 2024 Oct 21;7:238. doi: 10.1038/s42004-024-01293-1 (PMC11494096; doi:10.1038/s42004-024-01293-1)
Supplement: Supplementary file 1 — Supplementary Material [file 42004_2024_1293_MOESM1_ESM.pdf]

Supplementary material: $Ca_3[C_2O_5]_2[CO_3]$ is a pyrocarbonate which can be formed at p, T-conditions prevalent in the Earth's transition zone

Dominik Spahr[*,a], Lkhamsuren Bayarjargal[a], Maxim Bykov[b], Lukas Brüning[b], Pascal L. Jurzick[c], Yu Wang[a], Victor Milman[d], Keith Refson[e], Mohamed Mezouar[f], Björn Winkler[a]

[a]*Goethe University Frankfurt, Institute of Geosciences, Altenhöferallee 1, 60438 Frankfurt, Germany*
[b]*Goethe University Frankfurt, Institute of Inorganic and Analytical Chemistry, Max-von-Laue-Straße 7, 60438 Frankfurt, Germany*
[c]*University of Cologne, Institute of Inorganic Chemistry, Greinstraße 6, 50939 Cologne, Germany*
[d]*Dassault Systèmes BIOVIA, 22 Cambridge Science Park, Cambridge CB4 0FJ, United Kingdom*
[e]*ISIS Facility, Science and Technology Facilities Council, Harwell Campus, Chilton, Didcot, Oxon OX11, United Kingdom*
[f]*European Synchrotron Radiation Facility ESRF, 71 avenue des Martyrs, CS40220, 38043 Grenoble Cedex 9, France*

Supplementary Discussion

Synchrotron X-ray diffraction

Due to the identification of the unknown phases by Raman spectroscopy, we were able to perform synchrotron X-ray diffraction experiments in the regions where mainly Raman modes of one of the unknown phases have been observed in the DAC. After the data reduction the crystal structure was solved in the monoclinic space group $P2_1/n$ (No. 14) with $Z = 8$ (Table S 1).

Table S 1: Structural parameters of $Ca_3[C_2O_5]_2[CO_3]$ at 20(2) GPa from single crystal structure solution (ambient temperature) in comparison to DFT calculations (athermal limit).

	**Single Crystal**	**DFT (v.d.W.)**
**Crystal data**		
Crystal system	Monoclinic	
Space group	$P2_1/n$	
Chemical formula	$Ca_3[C_2O_5]_2[CO_3]$	
M_r	388.29	
a (Å)	8.020(4)	8.0626
b (Å)	8.8450(7)	8.9323
c (Å)	20.616(12)	20.7565
β (°)	96.18(6)	96.41
V (Å^3)	1453.9(11)	1485.49
Z	8	8
**Data collection**		-
F_{000}	1552	-
θ range (°)	1.82–18.73	-
measured reflections	3859	-
independent reflections	2104	-
reflections $I > 2\sigma(I)$	1455	-
R_{int}	0.018	-
		-
**Refinement**		-
$R[F^2 > 2\sigma(F^2)]$, $wR(F^2)$	0.078, 0.234	-
No. of reflections	2104	-
No. of parameters	199	-
No. of restraints	0	-
No. of constraints	0	-
$\Delta\rho_{max}$, $\Delta\rho_{min}$ (e Å^{-3})	1.64, -0.65	-

We found that the unknown phase is a mixed calcium carbonate-pyrocarbonate with $Ca_3[C_2O_5]_2[CO_3]$ composition. We reached a stable crystal structure refinement with a reasonable R-value (7.8%) and a sufficient reflection to parameter ratio (10.6:1). Table S 1 lists the crystallographic parameters of $Ca_3[C_2O_5]_2[CO_3]$ valid for 20(2) GPa in comparison to DFT calculations. The agreement between the experimental and calculated data is very good. In order to reduce the amount of parameters the atomic displacement parameters of the carbon and oxygen atoms were refined isotropically. The displacement parameters of the heavier calcium atoms were refined anisotropically. No constraints or restraints had been applied for the atomic positions or displacement parameters. The PLATON/checkCIF program does not suggest a higher space group symmetry or missing symmetry elements[1].

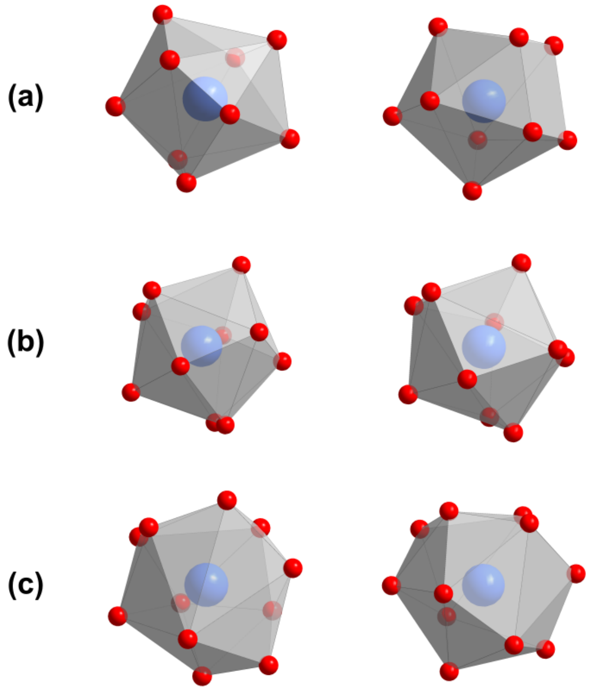

Figure S 1: Coordination polyhedra (grey) of a the Ca^{2+}-cations in the crystal structure of $Ca_3[C_2O_5]_2[CO_3]$: (a) $CN = 9$, (b) $CN = 10$ and (c) $CN = 11$.

*d.spahr@kristall.uni-frankfurt.de

In the crystal structure of $Ca_3[C_2O_5]_2[CO_3]$ the six calcium atoms are irregularly coordinated the by oxygen atoms. The coordination number of the calcium atoms (CN) ranges from 9 to 11 oxygen atoms (Fig. S 1). The Ca–O bond distances in the polyhedra are between 2.2 Å and 2.9 Å.

Bulk modulus of $Ca_3[C_2O_5]_2[CO_3]$

The p, V relation for $Ca_3[C_2O_5]_2[CO_3]$ was derived from DFT-based calculations. The calculations were carried out between 0 GPa and 35 GPa. One data-set was calculated without a v.d.W. correction and the second data-set was obtained using the TS-v.d.W. correction scheme[2]. We found that down to ≈ 10 GPa no significant difference between both theoretical data-sets can be observed (Fig. S 2). Below this pressure, the unit cell volume obtained without a v.d.W. correction is significantly too large.

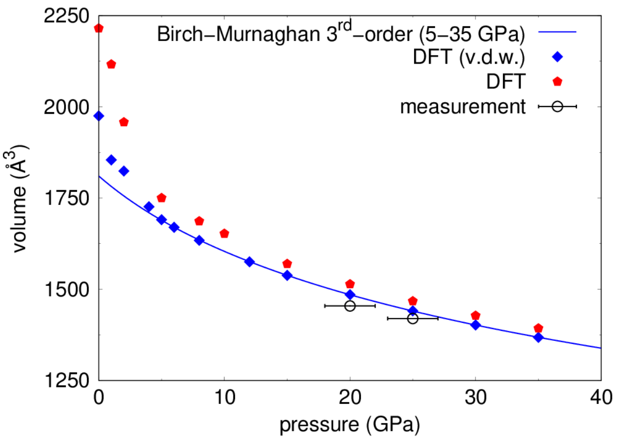

Figure S 2: A Birch-Murnaghan EoS was fitted to the unit cell volume of $Ca_3[C_2O_5]_2[CO_3]$ obtained from DFT-based calculations using the TS-v.d.W. correction scheme between 5–35 GPa. The results from the single crystal structure solutions are shown for comparison.

We found that the theoretical data between ambient pressure and 4 GPa cannot reasonably be described by an extrapolation of the high-pressure behavior. Hence, we decided to use the p, V data for pressures $\geq$ 5 GPa to obtain the theoretical bulk modulus (K_0) of $Ca_3[C_2O_5]_2[CO_3]$. We fitted a 3rd-order Birch-Murnaghan equation of states (EoS)[3,4] to the unit cell volume of the v.d.W. corected DFT-data between 5–35 GPa using the software package EOSFit7-GUI[5]. We obtained a bulk modulus of $K_0 = 60(1)$ GPa with $K_p = 5.2(1)$ and $V_0 = 1811(3)$ Å.

Decompression of $Ca_3[C_2O_5]_2[CO_3]$

We measured Raman spectroscopy during the decompression of the DAC. We found that at pressures $\geq$ 5 GPa the characteristic Raman modes of $Ca_3[C_2O_5]_2[CO_3]$, in the rection between 450–1200 cm^{-1}, are present. Figure S 3 shows the Raman spectrum of $Ca_3[C_2O_5]_2[CO_3]$ at 5(1) GPa in comparison with the Raman spectrum of CO_2-I (dry ice) measured in the same DAC. At ambient conditions only the Raman modes of the $Ca[CO_3]$ phase calcite were obtained in the gasket hole after pressure release (Fig. S 3).

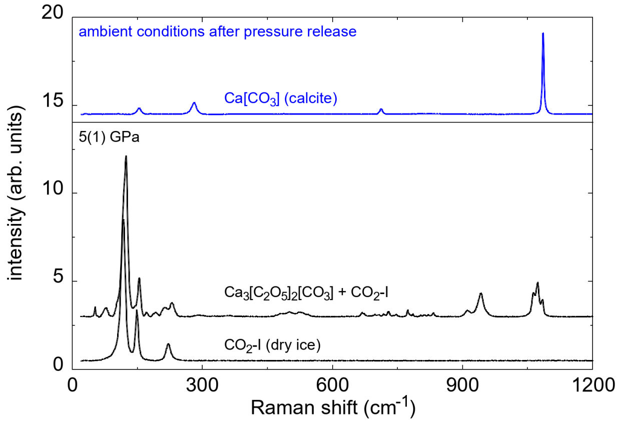

Figure S 3: Experimental Raman spectra of CO_2-I (dry ice) and of a $Ca_3[C_2O_5]_2[CO_3]$ + CO_2-I mixture after decompression of the DAC to 5(1) GPa (black). Raman spectrum of calcite ($Ca[CO_3]$) after decompressing the DAC to ambient conditions (blue).

Supplementary References

(1) Spek, A. L. Single-crystal structure validation with the program *PLATON*. *J. Appl. Cryst.* **2003**, *36*, 7–13, DOI: 10.1107/S0021889802022112

(2) Tkatchenko, A.; Scheffler, M. Accurate Molecular Van Der Waals Interactions from Ground-State Electron Density and Free-Atom Reference Data. *Phys. Rev. Lett.* **2009**, *102*, 073005, DOI: 10.1103/PhysRevLett.102.073005

(3) Murnaghan, F. The Compressibility of Media under Extreme Pressures. *Proc. Natl. Acad. Sci.* **1944**, *30*, 244–247, DOI: 10.1073/pnas.30.9.244

(4) Birch, F. Finite Elastic Strain of Cubic Crystals. *Phys. Rev.* **1947**, *71*, 809–824, DOI: 10.1103/PhysRev.71.809

(5) Gonzalez-Platas, J.; Alvaro, M.; Nestola, F.; Angel, R. *EosFit7-GUI*: a new graphical user interface for equation of state calculations, analyses and teaching. *J. Appl. Cryst.* **2016**, *49*, 1377–1382, DOI: 10.1107/S1600576716008050
